# Supplementary material for: Association of Diabetes with Greater Mid-Term Cognitive Decline After Carotid Surgery
Source: Biomedicines. 2025 Sep 7;13(9):2188. doi: 10.3390/biomedicines13092188 (PMC12467839; doi:10.3390/biomedicines13092188)
Supplement: Supplementary file 1 [file biomedicines-13-02188-s001.zip › biomedicines-3815608-supplementary.pdf]

Association of diabetes with greater mid-term cognitive decline after carotid surgery

Supplementary material

Á. D. Sándor[1], P. M. Sikos[2], G. Vavrinot[3], F. Kallinikos[4], Cs. Mánfai[5], M. Ifju[6], T. Kézi[7], Zs. Czinege[8], A. Szabó[9], Zs. Mihály[8], P. Sótonyi[8], A. Székely[1,10*]

[1] Department of Anesthesiology and Intensive Therapy, Semmelweis University, Budapest, Hungary

[2] Master's Program in Autonomous Systems, Faculty of Informatics, Eötvös Loránd University, Budapest, Hungary

[3] Department of Medicine, Université Laval, Québec, QC, Canada

[4] Department of Medicine, University of Patras, Greece

[5] Mihály Fazekas High School, Budapest, Hungary

[6] Doctoral School, Semmelweis University, Budapest, Hungary

[7] Genotech Ltd. Budapest, Hungary

[8] Department of Vascular and Endovascular Surgery, Semmelweis University, Budapest, Hungary

[9] Department of Anesthesiology and Intensive Care Unit Petz Aladár University Teaching Hospital, Győr, Hungary

[10] Department of Oxyology and Emergency Care, Faculty of Nursing Studies, Semmelweis University

Corresponding author

Andrea Székely, MD, PhD, FESAIC

Department of Anesthesiology and Intensive Therapy

Semmelweis University Budapest

Hungary

[*] szekely.andrea1@semmelweis.hu

Supplement

Diabetes medication	Number of patients (N)	Percentage of patients (%)
One oral antidiabetic active substance/hypoglycemic agent mediaction	20	54.05
Two or more oral antidiabetic active substances	8	21.62
Oral antidiabetic medication + insulin	4	10.81
Insulin	5	13.51

Supplementary Table S1. Summary of diabetic medications in the diabetic group

Hypoglycemic agent	Number of diabetic patients using the agent	Percentage of diabetic patients using the agent (%)
Biguanides	25	67.56
Sulfonylureas	8	21.62
DPP-4 Inhibitors	4	10.81
SGLT2 Inhibitors	2	5.40
GLP-1 Agonists	2	5.40

Supplementary Table S2. The applied hypoglycemic agents and their frequencies
DPP-4 inhibitors: dipeptidyl peptidase 4 inhibitors, GLP-1 agonists: glucagon-like peptide-1 receptor agonists
SGLT2 inhibitors: sodium-glucose cotransporter-2 (SGLT2) inhibitors

Characteristics	Change of the MoCA scores 3 months after the operation					Change of the MoCA scores one year after the operation				
	B	Std. Error	Beta	t	Sig.	B	Std. Error	Beta	t	Sig.
Age	0.001	0.027	-0.002	-0.016	0.987	0.028	0.036	0.084	0.784	0.435
Male sex	1.000	0.374	0.257	2.673	**0.009**	1.644	0.452	0.365	3.634	**0.001**
Median of the rSO2 in the preclamping period	0.039	0.020	0.192	1.969	0.054	0.049	0.025	0.205	1.937	0.052
Median of the lowest rSO2 during the cross-clamp period	0.098	0.015	0.548	6.592	**0.001**	0.132	0.017	0.637	7.663	**0.001**
Median of the degree of desaturation during the cross-clamp period	-0.142	0.015	-0.695	-9.711	**0.001**	-0.187	0.015	-0.807	-12.684	**0.001**
ASA score	0.121	0.381	0.032	0.318	0.751	0.117	0.466	0.027	0.252	0.802
Vascular-Possum score	-0.063	0.050	-0.124	-1.253	0.213	-0.012	0.065	-0.020	-0.181	0.857
Hypertension	-0.672	0.713	-0.093	-0.944	0.348	-0.272	0.889	-0.033	-0.305	0.761
Ischemic heart disease	-0.289	0.403	-0.071	-0.716	0.476	-0.062	0.504	-0.013	-0.124	0.902
COPD	-0.316	0.483	-0.065	-0.654	0.514	0.244	0.596	0.044	0.410	0.683
Diabetes mellitus (NIDDM + IDDM)	-0.904	0.392	-0.224	-2.309	**0.023**	-1.129	0.485	-0.243	-2.327	**0.022**
Preoperative fasting blood sugar level	-0.246	0.152	-0.172	-1.615	0.110	-0.226	0.180	-0.143	-1.255	0.213
Depression or mood disorder	-0.948	0.496	-0.187	-1.914	0.058	-0.892	0.651	-0.146	-1.370	0.174
Previous stroke	0.358	0.459	0.077	0.780	0.437	0.462	0.638	0.078	0.724	0.471
Hyperlipidemia	-0.146	0.383	-0.038	-0.381	0.704	-0.182	0.481	-0.041	-0.378	0.706
Statin use	0.049	0.393	0.012	0.124	0.901	0.168	0.494	0.037	0.340	0.735
Peripheral arterial disease	-0.441	0.451	-0.097	-0.978	0.331	-0.971	0.565	-0.182	-1.719	0.089
Smoking (current)	-0.399	0.416	-0.095	-0.960	0.339	-0.995	0.506	-0.208	-1.969	0.052
Alcohol consumption	0.749	0.592	0.125	1.264	0.209	0.508	0.756	0.072	0.671	0.504
BMI	-0.052	0.042	-0.123	-1.247	0.215	-0.009	0.053	-0.018	-0.163	0.871
Education (yrs)	-0.012	0.077	-0.016	-0.160	0.873	0.115	0.099	0.124	1.159	0.250
Time of the cross-clamp	-0.063	0.025	-0.283	-2.520	**0.014**	-0.037	0.030	-0.151	-1.214	0.229
Use of shunt	-0.655	0.421	-0.153	-1.555	0.123	-1.547	0.514	-0.309	-3.009	**0.003**
Operated side	0.282	0.382	0.073	0.736	0.463	0.239	0.481	0.053	0.497	0.621
Degree of the stenosis on the operated side	-0.015	0.025	-0.058	-0.581	0.563	-0.029	0.033	-0.095	-0.885	0.378
Degree of the stenosis on the contralateral. side	-0.003	0.006	-0.052	-0.521	0.604	-0.011	0.008	-0.161	-1.509	0.135
Anatomy of Circle of Willis	-0.669	0.425	-0.161	-1.576	0.118	-0.667	0.515	-0.144	-1.295	0.199

Supplementary Table S3. Results of the linear regression analysis for all of the variables in relation to the change of the MoCA scores

OR: odds ratio, C.I.: confidence interval, ASA score: American Society of Anesthesiologists physical status classification, BMI: body mass index, COPD: chronic obstructive pulmonary disease, HbA1c: hemoglobin A1c, IDDM: insulin dependent diabetes mellitus, MAP: mean arterial pressure, NIDDM: noninsulin dependent diabetes mellitus, rSO_2: regional cerebral oxygen saturation, Vascular-POSSUM: Vascular-Physiological and Operative Severity Score for the enUmeration of Mortality and Morbidity

*analyzed among diabetic patients

Characteristics	Change of the MoCA scores 3 months after the operation					Change of the MoCA scores one year after the operation				
	B	Std. Error	Beta	t	Sig.	B	Std. Error	Beta	t	Sig.
Male sex	0.406	0.354	0.106	1.146	0.256	0.942	0.287	0.209	3.281	**0.002**
Median of the rSO2 in the preclamping period	0.277	0.183	1.325	1.514	0.135	0.149	0.133	0.628	1.117	0.267
Median of the lowest rSO2 during the cross-clamping period	-0.294	0.213	-1.510	-1.380	0.172	-0.169	0.160	-0.819	-1.058	0.293
Median of the degree of desaturation during the cross-clamping period	-0.332	0.146	-1.509	-2.277	**0.026**	-0.280	0.109	-1.206	-2.555	**0.013**
Diabetes mellitus	-0.188	0.381	-0.043	-0.494	0.623	-0.865	0.287	-0.186	-3.010	**0.003**
Depression or mood disorder	-0.644	0.447	-0.123	-1.442	0.154	-	-	-	-	-
Time of the cross-clamping period	-0.051	0.019	-0.227	-2.668	**0.010**	-	-	-	-	-
Use of shunt	-	-	-	-	-	-0.530	0.319	-0.106	-1.663	0.100

Supplementary Table S4. Results of the multiple linear regression analysis for all of the variables in relation to the change of the MoCA scores

OR: odds ratio, C.I.: confidence interval, ASA score: American Society of Anesthesiologists physical status classification, BMI: body mass index, COPD: Chronic obstructive pulmonary disease, HbA1c: hemoglobin A1c, IDDM: insulin dependent diabetes mellitus, MAP: mean arterial pressure, NIDDM: noninsulin dependent diabetes mellitus, rSO_2: regional cerebral oxygen saturation, Vascular-POSSUM: Vascular-Physiological and Operative Severity Score for the enUmeration of Mortality and Morbidity

*analyzed among diabetic patients

Characteristics	Long-term mortality and cardiovascular and cerebrovascular complications			
	OR	95% C.I.		Significance
		Lower	Upper	
Age	1.042	0.971	1.117	0.255
Male	0.186	0.061	0.565	**0.003**
Median of the rSO2 in the preclamping period	0.945	0.893	1.000	0.051
Median of the lowest rSO2 during the cross-clamping period	0.950	0.908	0.994	**0.025**
Median of the degree of desaturation during the cross-clamping period	1.032	0.986	1.081	0.176
Change of the MoCA scores at three months after surgery	0.728	0.571	0.928	0.010
Deterioration of cognitive function demonstrated by the MoCA test	4.900	1.724	13.927	**0.003**
ASA score	1.450	0.547	3.848	0.455
Vascular-Possum score	1.037	0.914	1.176	0.571
Score of Blecha et al.	1.414	1.206	1.658	**0.001**
Hypertension	0.341	0.098	1.194	0.092
Ischemic heart disease	1.384	0.536	3.572	0.502
COPD	0.819	0.237	2.831	0.753
Diabetes mellitus treated with oral hypoglycemic medications	3.617	1.435	9.120	**0.006**
Diabetes mellitus treated with insulin	0.975	0.224	4.240	0.973
Diabetes mellitus	3.221	1.248	8.313	**0.016**
Preoperative fasting blood sugar level	1.284	0.936	1.762	0.121
Previous stroke	1.962	0.725	5.305	0.184
Hyperlipidemia	1.885	0.697	5.101	0.212
Depression or mood disorders	1.808	1.853	12.476	0.701
Peripheral arterial disease	2.480	0.943	6.518	0.065
Smoking (current)	2.160	0.832	5.607	0.113
Alcohol consumption	1.062	0.243	4.644	0.936
BMI <20	2.528	0.335	19.088	0.369
BMI >30	0.327	0.075	1.429	0.137
Education (yrs)	0.951	0.779	1.161	0.621
Time of the cross-clamp	0.996	0.929	1.067	0.905
Degree of the stenosis on the operated side	1.025	0.958	1.096	0.478
Degree of the stenosis on the contralateral side	1.013	0.997	1.028	0.104
Anatomy of Circle of Willis	2.073	0.591	7.276	0.255

Supplementary Table S5. Results of Cox regression analysis for all of the variables in relation to mortality and long-term cardiovascular and cerebrovascular complications

OR: odds ratio, C.I.: confidence interval, ASA score: American Society of Anesthesiologists physical status classification, BMI: body mass index, COPD: chronic obstructive pulmonary disease, HbA1c: hemoglobin A1c, MoCA: Montreal Cognitive Assessment, rSO_2: regional cerebral oxygen saturation, Vascular-POSSUM: Vascular-Physiological and Operative Severity Score for the enUmeration of Mortality and Morbidity
